# Technology and Rheological Properties of Warm Asphalt Rubber Based on an Ultra-Warm Mix Additive (UWM)–Sasobit Composite System

**DOI:** 10.3390/polym18010007

**Published:** 2025-12-19

**Authors:** Song Xu, Longxiang Zhao, Shishui Liulin, Xiangjie Niu, Xiaojuan Jia, Hui Cai

**Affiliations:** 1 College of Civil Engineering, Fuzhou University, Fuzhou 350108, China; z.longxiang@foxmail.com (L.Z.); xiangjieniu@163.com (X.N.); 2 Fujian Transportation Development High-Tech Co., Ltd., Fuzhou 350004, China; ruasll@163.com; 3 Fujian Provincial Expressway Technology Consulting Co., Ltd., Fuzhou 350108, China; 4 Fujian Expressway Maintenance Engineering Co., Ltd., Fuzhou 350019, China; zywzhpljh88@163.com

**Keywords:** asphalt rubber (AR), warm asphalt rubber (WAR), warm mix asphalt, orthogonal experimental design, Ultra-Warm Mix additive (UWM), Sasobit, rheological properties

## Abstract

To address the challenges of decarbonization in the global transportation sector and disposal of waste tires, warm asphalt rubber (WAR) with low viscosity and high performance was prepared. In particular, the preparation and rheological behavior of WAR incorporating composite warm mix systems at relatively high crumb rubber contents have not been thoroughly documented. In this study, WAR prepared under such conditions was systematically examined. A five-factor, three-level segmented orthogonal experimental design (OED) was employed to investigate the effects of preparation parameters on hot mix asphalt rubber (AR) properties. Based on the optimized AR formulation, a composite warm mix system combining Ultra-Warm Mix additive (UWM) and Sasobit was developed, and control groups containing 5% UWM only and 1.5% Sasobit only were prepared for comparison. Conventional physical tests together with rheological characterization, including Dynamic Shear Rheometer (DSR), Multiple Stress Creep Recovery (MSCR), and Bending Beam Rheometer (BBR) tests, were conducted to evaluate the high- and low-temperature performance of WAR. Results show that the optimal preparation process consisted of aromatic oil content 5%, crumb rubber content 30%, shear temperature 220 °C, shear time 120 min, and reaction time 90 min. The composite warm mix system notably enhanced WAR performance, with the WAR-5U1.5S group exhibiting the most balanced properties. A marked reduction in rotational viscosity was achieved while maintaining a stable softening point, and satisfactory ductility and elastic recovery were also retained. DSR and MSCR tests confirmed improved high-temperature deformation resistance, an increase in percent recovery R, and a decrease in non-recoverable creep compliance J_nr_. BBR test further verified that the composite system maintained good low-temperature cracking resistance, meeting all specification requirements. Overall, these results indicate that, compared with the optimized AR, WAR can reduce mixing viscosity without sacrificing rutting or cracking performance, while alleviating the limitations observed for single warm mix additives. This study provides essential technical support for promoting WAR that integrates low-carbon construction with superior pavement performance.

## 1. Introduction

Asphalt, with its excellent adhesion, durability, and waterproofing properties, is a key material in road construction. Although traditional hot mix asphalt (HMA) is widely used due to its balanced performance, its production and paving require heating to above 150 °C, consuming large amounts of fossil fuels and emitting CO_2_, NO_x_, and volatile organic compounds (VOCs), which contradicts global transportation decarbonization goals [1,2]. Meanwhile, the continuous expansion of the automotive industry has generated a massive amount of waste tires. By 2023, the global annual production of waste tires reached approximately 1.5 billion, with tire production growing at an annual rate of about 4% [3]. Current mainstream disposal methods such as landfilling and incineration occupy valuable land resources and release toxic gases including polycyclic aromatic hydrocarbons, posing serious risks to ecosystems and human health [4,5,6]. Under this dual pressure, the development of asphalt modification technologies that reconcile low-carbon construction and resource recycling has become urgent.

The use of crumb rubber derived from waste tires for asphalt modification has attracted attention for its dual environmental and performance benefits, offering an effective recycling route while meeting environmental requirements [7]. Huang et al. [8] found that when crumb rubber is incorporated into hot asphalt, it swells by absorbing oils under thermal and mechanical action to form a three-dimensional elastic network, which can significantly improve pavement performance at low cost and reduce environmental pollution from rubber products. Zhang et al. [9] used Dynamic Shear Rheometer (DSR) and Multiple Stress Creep Recovery (MSCR) tests to confirm that the incorporation of crumb rubber significantly enhances the high-temperature rutting resistance of base asphalt. Lyu et al. [10] pointed out that crumb rubber modification can mitigate aging-induced hardening and contribute to longer pavement service life from a sustainability perspective.

Meanwhile, the production process of asphalt rubber (AR) has also attracted much attention. Guo et al. [11] indicated that the addition of bio-oil enhanced the anti-fatigue properties and positively influenced the low-temperature properties of SBS and crumb rubber-modified asphalt. Li et al. [12] optimized the dry modification process of rubber and plastic in an asphalt mixture by the orthogonal experiments, thereby achieving durable and sustainable pavement construction. Zhao et al. [13] used the orthogonal experimental design (OED) method to determine the priority order of the influencing factors on two novel asphalt rubber pellets and asphalt desulfurized rubber pellets, and further optimized the mixing technology of asphalt mixtures containing direct injection modifiers. Moreover, the wet process has become mainstream due to its high efficiency and stable quality, and the AR produced by the wet process shows superior high-temperature stability and low-temperature cracking resistance compared with the dry process [14]. Factors such as rubber powder gradation, preparation temperature, and preparation time significantly influence AR performance, providing opportunities for process optimization to meet different engineering needs [15]. Dong et al. [16] reported from Bending Beam Rheometer (BBR) tests that increasing preparation temperature and time improves the low-temperature cracking resistance of AR, while optimized processing reduces rubber particle size and achieves a more uniform distribution. However, conventional AR preparation still faces a core bottleneck, namely that crumb rubber requires devulcanization and desulfurization at temperatures above 200 °C, which increases energy consumption and carbon emissions and accelerates asphalt aging [17]. Controlling organic emissions from crumb rubber-modified asphalt is also necessary for clean application [18].

The emergence of warm mix asphalt (WMA) technology provides an effective path to reduce the construction temperature of AR. Among them, the organic warm mix additive, Sasobit, is widely used in warm asphalt rubber (WAR) research because of its strong adaptability and pronounced viscosity reduction [19]. Yang et al. [20] investigated the rheological properties of binders containing two types of WMA additives, Deurex and Sasobit, and the results showed that the Sasobit-modified asphalt binders exhibit a slightly higher storage modulus and complex modulus but a lower phase angle than the Deurex-modified asphalt. Ghazi et al. [21] systematically investigated the rheology of crumb rubber-modified warm mix asphalt and showed that Sasobit-modified asphalt binders exhibited superior resistance to aging and rutting. Sasobit reduces binder viscosity by regulating asphalt colloid structure while raising the softening point, thereby improving both the workability and high-temperature stability of AR [22,23]. However, the application of Sasobit has obvious performance shortcomings. The microcrystalline wax that strengthens the high-temperature performance also limits molecular chain flexibility, which reduces ductility and increases the low-temperature brittleness risk [24]. This performance imbalance of reducing viscosity but impairing ductility makes it difficult for single Sasobit modification to meet the comprehensive requirements of WAR for both high- and low-temperature performance. Chemical warm mix additives have less impact on conventional binder indicators but exhibit weaker viscosity reduction, and in some cases even increase viscosity [25]. Gui et al. [26] evaluated warm mix crumb rubber binders with different chemical warm mix additives and found that the additives effectively reduce viscosity but may slightly decrease high-temperature stiffness. At the mixture scale, Miguel et al. [27] demonstrated that asphalt mixtures incorporating waste crumb rubber and a surfactant-based warm mix additive improved the mechanical requirements while lowering production temperature. Guo et al. [28] showed that when high crumb rubber contents are used, appropriate warm mix additives are needed to control viscosity while maintaining balanced rheological properties. A single warm mix additive cannot simultaneously meet the core needs of WAR for efficient viscosity reduction, high-temperature deformation resistance, and low-temperature brittleness resistance. Therefore, a composite warm mix system is required to exploit the complementary advantages of different additives and address the limitations observed for single materials. However, the current research on the synergistic mechanism of composite warm mix systems is insufficient, optimal additive ratios and preparation procedures for WAR remain unclear, and the performance evaluation relies too heavily on conventional physical indicators with insufficient rheological data. These limitations hinder the establishment of clear relationships between processing parameters and material properties, and restrict the engineering application of WAR.

Based on the above situation, this study aims to prepare WAR with low viscosity and high performance, specifically focusing on two aspects of work. On one hand, a segmented orthogonal experimental design (OED) was adopted, combined with range analysis, analysis of variance (ANOVA), and weighted scoring methods, to determine the optimal preparation process for AR. Moreover, the warm mix additives, namely UWM and Sasobit, were used to prepare WAR. Physical property tests and rheological tests using DSR, MSCR, and BBR were conducted to comprehensively assess workability and high- and low-temperature performance. Thus, the study links a segmented OED for AR preparation with a composite UWM–Sasobit warm mix system and evaluates its ability to reduce viscosity while maintaining balanced rheological performance.

## 2. Materials and Methods

### 2.1. Materials

The 70# base asphalt used for modification was supplied by China National Petroleum Corporation (Beijing, China), and its main properties are shown in Table 1. Crumb rubber with a mesh size of 80 was produced by mechanical grinding at ambient temperature and provided by Huayi Rubber Co., Ltd. (Dujiangyan, China); its technical indicators are presented in Table 2. Aromatic oil was used as a modifier aid because its high aromatic content effectively dissolves rubber agglomerates, reduces system viscosity, and improves flexibility, while enhancing adhesion to aggregates by increasing the colloidal fraction; its technical indicators are shown in Table 3. Styrene–butadiene–styrene (SBS) modifier YH-791, a linear type with a block ratio (S/B) of 30/70, produced by Baling Petrochemical (Yueyang, China), was used at a content of 2% to optimize the storage stability and low-temperature crack resistance of the asphalt rubber.

The Ultra-Warm Mix additive (UWM) was a laboratory-synthesized warm mix additive prepared using waste rubber and plastic as raw materials. It was formulated as a composite system including rubber-derived components and surfactant components. The detailed formulation and proportions are not disclosed at this stage because a patent application is pending. Sasobit, a long-chain alkane microcrystalline wax produced by the Fischer–Tropsch synthesis of coal-based syngas, was selected for its ability to significantly enhance high-temperature performance. The properties of both warm mix additives are shown in Table 4.

### 2.2. Orthogonal Experimental Design

To investigate the effects of different preparation parameters on the properties of AR, a five-factor, three-level segmented orthogonal experimental design (OED) was employed. OED is a statistical method for studying multiple factors and objectives simultaneously, allowing analysis of combined effects through a limited number of tests. Aromatic oil content (A), crumb rubber content (B), shear temperature (C), shear time (D), and reaction time (E) were selected as the investigated factors. The levels for each factor were set as follows: aromatic oil 4%, 5%, 6%; crumb rubber was divided into low-content group (10%, 15%, 20%) and high-content group (25%, 30%, 35%); shear temperature 180 °C, 200 °C, 220 °C; shear time 60 min, 90 min, 120 min; and reaction time 30 min, 60 min, 90 min. The overall range of crumb rubber content from 10% to 35% was selected to cover the typical dosage used in engineering practice. At lower contents, the viscosity of AR is relatively low, but the improvement in high-temperature performance and elasticity may be insufficient and the effective utilization of crumb rubber is limited. At higher contents, more crumb rubber can be used to enhance stiffness and elasticity and to reduce the binder cost, but the viscosity increases markedly and construction becomes more difficult. Therefore, both low-content and high-content AR needed to be considered in the orthogonal experimental design, and the two dosage ranges were arranged and analyzed separately in the segmented orthogonal experimental design.

Aromatic oil content was set at 4–6% to ensure adequate viscosity reduction and rubber dispersion while avoiding excessive softening point decrease and increased cost at higher contents. Crumb rubber content, the dominant factor influencing rotational viscosity, material cost, and rubber utilization, was divided into low-content (10–20%) and high-content (25–35%) groups. In the preliminary orthogonal arrangement, crumb rubber content was first treated as a single factor with six levels from 10% to 35% at 5% intervals. Range analysis of the test results showed that the range of crumb rubber content was consistently much larger than those of the other factors for all performance indicators, so the influence of the other factors could not be evaluated clearly. For this reason, the low-content and high-content groups were analyzed separately in the segmented orthogonal experimental design, and range analysis was carried out for each group. This approach improves the resolution of the other factors under different crumb rubber dosage ranges. The shear temperature levels of 180 °C, 200 °C, and 220 °C were selected to balance energy consumption with the requirements for rubber swelling and dispersion while preventing asphalt aging. Shear time levels of 60, 90, and 120 min were chosen to ensure sufficient rubber particle breakdown and system homogenization while allowing evaluation of potential performance gains. The selection of reaction time was based on the compatibility between the modifier and asphalt as well as the time cost, and the levels of 30, 60, and 90 min ensured a balance between reaction sufficiency and production efficiency.

Within this segmented orthogonal experimental design, the main effects of the five factors are obtained from the orthogonal arrangement. Explicit interaction terms between factors are not added in order to keep the number of test groups within a reasonable range. In the orthogonal table, each of the first nine groups corresponds to one of the last nine groups with the same levels of the other factors and a different level of crumb rubber content. By comparing the performance differences within these pairs, the combined influence of crumb rubber content and the other factors can be examined, and possible interactions are reflected indirectly in the trends of viscosity, softening point, and rheological indices under different factor combinations. These combined effects are discussed in the Results and Discussion section, rather than being separated as independent interaction factors in the design matrix. The orthogonal experimental design is shown in Table 5.

The rotational viscosity at 145 °C, penetration, softening point, ductility, and elastic recovery were selected as evaluation indicators. The range analysis was used to determine the sensitivity of each factor to performance indices. This method calculates the mean range of each factor, where a higher range R indicates a stronger influence on the target index. The detailed formulas for R are shown in Equations (1) and (2).

(1) $$k_{ij}=\frac{K_{ij}}{r}$$

(2) $$R=max\left\{k_{ij}\right\}-min\left\{k_{ij}\right\}$$

where $K_{ij}$ is the sum of indicator values for the j-th factor at the i-th level. r is the number of occurrences of the j-th factor at the i-th level. $k_{ij}$ is the average value of $K_{ij}$. R is the range, where a larger R value indicates a greater influence on the evaluation indicator.

In addition to range analysis, one-way analysis of variance (ANOVA) was used to further evaluate the statistical significance of the preparation factors. For each performance index, the orthogonal experimental results were grouped according to the levels of a given factor, and the between-group and within-group variations were calculated. The ANOVA statistics are shown in Equations (3)–(9).

(3) $${SS}_{between}=\sum_{i=1}^{k}{n_{i}\left({\bar{y}}_{i}-\bar{y}\right)}^{2}$$

(4) $${SS}_{within}=\sum_{i=1}^{k}{\sum_{j=1}^{n_{i}}\left(y_{ij}-{\bar{y}}_{i}\right)}^{2}$$

(5) $${df}_{between}=k-1$$

(6) $${df}_{within}=N-k$$

(7) $${MS}_{between}=\frac{{SS}_{between}}{{df}_{between}}$$

(8) $${MS}_{within}=\frac{{SS}_{within}}{{df}_{within}}$$

(9) $$F=\frac{{MS}_{between}}{{MS}_{within}}$$

where $y_{ij}$ is the value of the response index for the j-th test at the i-th level of a given factor, ${\bar{y}}_{i}$ is the mean value of the response index at the i-th level, and $\bar{y}$ is the overall mean value of the response index for all tests. k is the number of levels of the factor, $n_{i}$ is the number of tests at the i-th level, and N is the total number of tests. ${SS}_{between}$ and ${SS}_{within}$ are the between-group and within-group sums of squares, respectively. ${df}_{between}$ and ${df}_{within}$ are the corresponding degrees of freedom, ${MS}_{between}$ and ${MS}_{within}$ are the between-group and within-group mean squares, respectively, F is the F statistic used to assess the significance of the factor, and p denotes the significance level. A significance level of 0.05 was adopted in this study.

Through segmented range analysis and single-index analysis, the optimal parameter combination for both low- and high-content groups was identified. A weighted scoring method was then used to comprehensively evaluate all test groups and optimized schemes. The weights assigned to each performance indicator were as follows: rotational viscosity 40%, penetration 10%, softening point 30%, ductility 10%, and elastic recovery 10%.

Weight allocation was determined based on engineering requirements, preliminary test results, and performance optimization priorities. Rotational viscosity was assigned the highest weight because high rubber content markedly increases viscosity, limiting processing and construction feasibility. Softening point was given a 30% weight due to the need for enhanced high-temperature resistance in systems without warm mix additives. Preliminary tests showed that penetration, ductility, and elastic recovery all remained within acceptable ranges and exhibited limited sensitivity to further improvement; therefore, each was assigned a weight of 10%.

The optimized preparation process was determined based on performance and cost-effectiveness. The weight scoring equations are presented in Equations (10)–(14). The evaluation ranges were as follows: rotational viscosity 0–6 Pa·s, penetration 40–80 (0.1 mm), softening point 50–70 °C, ductility 10–25 cm, and elastic recovery 70–85%.

Rotational viscosity:

(10) $$\begin{array}{c} S_{Rotational\;viscosity}=40\times \left(1-\frac{Rotational\;viscosity}{6}\right) \end{array}$$

Penetration:

(11) $$\begin{array}{c} \begin{array}{c} S_{Penetration}=10\times \left(1-\frac{\left|Penetration-60\right|}{80-40}\right) \end{array} \end{array}$$

Softening point:

(12) $$S_{Softening\;point}=30\times \frac{Softening\;point-50}{70-50}$$

Ductility:

(13) $$\begin{array}{c} S_{Ductility}=10\times \frac{Ductility-10}{25-10} \end{array}$$

Elastic recovery:

(14) $$S_{Elastic\;recovery}=10\times \frac{Elastic\;recovery-70}{85-70}$$

Through implementation of these weighted scoring equations and indicator ranges, multidimensional performance indicators can be transformed into a unified quantitative scoring system. This framework clarified optimization direction and boundary limits, emphasized the central importance of viscosity and softening point, and effectively avoided conflicts among single-index optimizations. It provided a scientific and quantifiable basis for integrated analysis of orthogonal experimental results, process optimization, and cost–benefit evaluation.

### 2.3. Preparation of Warm Mix Rubber Composite-Modified Asphalt

The high-speed shearing was carried out using a laboratory high-speed shear mixer manufactured by Wuxi Petroleum Instrument Equipment Co., Ltd. (Wuxi, China), equipped with a high-speed shear head. A JJ-1 electric stirrer (Haijiangxing Instrument Co., Ltd., Shanghai, China) was used for low-speed mixing. Heating was provided by an electric furnace, and the preparation temperature was controlled using an intelligent temperature controller (ZNHW-II, Shanghai Biaohe Instrument Co., Ltd., Shanghai, China) throughout the mixing and shearing procedures. Since the power consumption was not recorded during preparation, shear energy was not calculated, and process consistency was ensured by controlling the batch mass, rotor speed, and temperature profile.

The preparation of WAR was based on the optimal parameters determined for AR. The procedure was as follows: 300 g base asphalt was heated in an oven at 160 °C until melted and fluid. The optimal content of aromatic oil was then added and stirred at 160 °C and 600 r/min for 15 min. The temperature was raised to the optimal shear temperature, and the optimal contents of crumb rubber and 2% SBS were added gradually while stirring at 600 r/min for 15 min to complete preliminary mixing. The mixture was then subjected to high-speed shearing at 3000 r/min under the optimal shear temperature for the optimal shear time. Finally, it was allowed to swell and react in an oven at 180 °C to obtain the optimized rubber-modified asphalt.

In this study, UWM was used as the base additive, and based on the viscosity–temperature curve results of preliminary tests, three dosage levels, 4%, 5%, and 6%, were set. To compensate for potential high-temperature performance loss caused by UWM, 1.5% Sasobit was added simultaneously to form a composite modification system. In addition, two control groups were prepared with 5% UWM only and 1.5% Sasobit only to verify the synergistic effect of the composite system. The UWM warm mix additive at the specified concentrations was preheated at 130 °C until molten and added together with 1.5% Sasobit into the AR cooled to 160 °C. The mixture was sheared at 160 °C and 3000 r/min for 30 min to obtain homogeneous WAR. The abbreviations and formulations of each asphalt binder are listed in Table 6.

**Figure 1:**
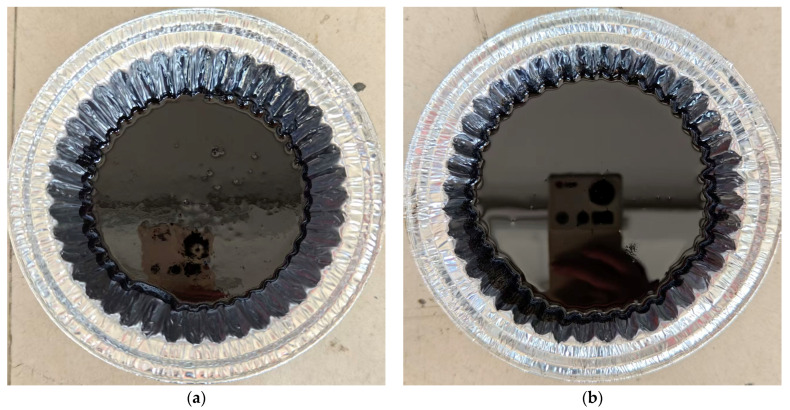
Appearance of AR and a representative WAR binder after preparation: (**a**) AR; (**b**) WAR.

A photograph of the prepared AR and a representative WAR binder is shown in Figure 1 to illustrate the appearance of the binders after preparation. The WAR binder appears more uniform and visually less viscous than AR, which is consistent with its reduced rotational viscosity.

### 2.4. Testing Methods

#### 2.4.1. Physical Property Tests

The physical property tests were used to evaluate the physical properties of binders as per JTG E20-2011 [29], including the rotational viscosity test, the penetration test, the softening point test, the ductility test, and the elastic recovery test. For rotational viscosity, the base asphalt was tested at both 135 °C and 145 °C, whereas the modified binders were measured at 145 °C, which was used as the evaluation index in the orthogonal analysis and in the comparison between AR and WAR.

#### 2.4.2. Rheological Property Tests

Temperature sweep test was employed using Dynamic Shear Rheometer (DSR) with 25 mm parallel plates (1 mm gap) at a frequency of 1.5 Hz in strain-controlled mode with a constant shear strain amplitude of 1%. The test was taken from 30 °C to 100 °C at a heating rate of 1 °C/min to determine complex modulus $G^{*}$, phase angle δ, and rutting factor $G^{*}/\sin\delta$. Binder specimens for DSR testing were prepared by pouring hot binder onto the center of the lower plate, then lowering the upper plate to a 1 mm gap. After the gap was stabilized for about 5 min, the excess binder was trimmed so that the edge was flush with the plate. Before each test, the specimen was conditioned at the target temperature for 10 min to reach thermal equilibrium.

The Multiple Stress Creep Recover (MSCR) test was conducted at 64 °C using 25 mm plates. Ten cycles of stress loading 0.1 kPa and 3.2 kPa were applied, each consisting of 1 s loading and 9 s recovery. The key parameters, percent recovery R, and non-recoverable creep compliance J_nr_ were calculated. The same plate geometry, specimen preparation procedure, and 1 mm gap as in the temperature sweep test were used, and each specimen was conditioned at 64 °C for 10 min before loading.

The Bending Beam Rheometer (BBR) test was performed at −12 °C, −18 °C, and −24 °C on asphalt samples. A load of 980 mN was applied for 60 s, and the creep stiffness S and creep rate m-value were recorded. The requirement of S ≤ 300 MPa and m-value ≥ 0.3 at 60 s must be met. BBR specimens were prepared in aluminum beam molds with dimensions of 127 mm × 12.7 mm × 6.35 mm. Before demolding, the molds containing the specimens were cooled at −5 °C for no more than 5 min to avoid deformation. After demolding, the beams were immediately placed in a temperature-controlled bath at the test temperature and conditioned for 60 min before loading.

In summary, the experimental program consists of three main stages. First, AR is produced by the wet process and a segmented OED is used to study the influence of five preparation parameters, aromatic oil content, crumb rubber content, shear temperature, shear time, and reaction time, on the physical properties of AR, including rotational viscosity at 145 °C, penetration, softening point, ductility, and elastic recovery. Second, the optimized AR obtained from the OED is used as the base binder to prepare a series of WAR samples with different warm mix formulations, including the UWM-only binder, the Sasobit only binder, and composite UWM–Sasobit binders with 30% crumb rubber content. Third, these binders are characterized by physical tests and rheological tests including DSR temperature sweep, MSCR, and BBR to determine their high- and low-temperature performance. This framework allows the relationships between preparation parameters, warm mix formulations, and the final properties of the UWM–Sasobit-based WAR and the control binders to be evaluated.

## 3. Results

### 3.1. Orthogonal Experimental Results

The core performance data from the orthogonal experiments are presented in Table A1. Eighteen sets of test results were obtained and divided into low and high crumb rubber content groups, providing a foundational dataset for subsequent range analysis and process optimization.

The range analysis results for the low crumb rubber content group are shown in Table A2. Among all factors, crumb rubber content B is the dominant factor influencing rotational viscosity. For softening point, aromatic oil content A has the greatest effect, followed by crumb rubber content B. Ductility is primarily controlled by shear time D, while elastic recovery is most affected by shear temperature C. Considering the optimization objectives of minimizing viscosity and maximizing softening point, ductility, and elastic recovery, along with maintaining moderate penetration, the optimal combination is determined based on weighted analysis. For rotational viscosity, although B1 results in low viscosity, its softening point and ductility are relatively low, failing to meet pavement performance requirements. In contrast, B3 achieves higher softening point and ductility while maintaining viscosity at 2.18 Pa·s, within acceptable construction limits, thus balancing workability and road performance. Consequently, crumb rubber content B3 is selected. Aromatic oil content A2 balances softening point and cost, shear temperature C2 balances penetration and energy consumption, shear time D3 optimizes ductility and elastic recovery, and reaction time E1 controls viscosity. The optimal combination for the low-content group is determined as K_1_: A2B3C2D3E1, i.e., aromatic oil content 5%, crumb rubber 20%, shear temperature 200 °C, shear time 120 min, and reaction time 30 min.

The range analysis results for the high crumb rubber content group are shown in Table A3. Crumb rubber content B has the most significant effect on rotational viscosity, with its k-value increasing markedly with content and directly determining the feasibility boundary for construction. Shear temperature C most strongly affects penetration, with C3 best matching consistency requirements. Reaction time E significantly affects softening point, with E3 yielding the highest value. Shear time D has a pronounced effect on ductility, with D3 achieving the highest ductility. Shear temperature C and reaction time E contribute substantially to elastic recovery, both achieving maximum recovery at C1 and E3. Considering the weighted priority of indicators, crumb rubber content B5 is selected to balance viscosity and rubber utilization. Thus, the optimal combination for the high-content group is determined as K_2_: A2B5C3D3E3, i.e., aromatic oil 5%, crumb rubber 30%, shear temperature 220 °C, shear time 120 min, and reaction time 90 min.

To further quantify the influence of each factor on the key performance indicators, one-way ANOVA was carried out based on the orthogonal test results. As summarized in Table 7, crumb rubber content B exhibits the largest F values for both rotational viscosity and softening point, with p < 0.001, indicating that it is the dominant factor over the full crumb rubber content range of 10–35%. In contrast, aromatic oil content A, shear temperature C, shear time D, and reaction time E show small F values and p > 0.05, suggesting that their effects on viscosity and softening point are statistically insignificant within this design and mainly serve as secondary tuning parameters. These ANOVA results are consistent with the range analysis and confirm that crumb rubber content is the primary parameter governing the viscosity and high-temperature properties of AR.

In addition, to clarify the rationale for the segmented orthogonal experimental design, one-way ANOVA for factor B is conducted separately for the low-content group (groups 1–9, 10–20%) and the high-content group (groups 10–18, 25–35%), as shown in Table 8. In the low-content group, crumb rubber content has a statistically significant effect on rotational viscosity, with an F value of 12.29 and a corresponding p value of 0.008, which is lower than the significance level of 0.05. Its effect on softening point in this group is not significant, as the p value is 0.332. In the high-content group, crumb rubber content still shows a clear influence trend on rotational viscosity, with an F value of 4.04 and a p value of 0.078, indicating a weaker effect within this narrower range that does not reach the 0.05 significance level. For softening point, the p values in both subranges are greater than 0.05, showing that the influence of crumb rubber content is not statistically significant within either range. These results indicate that the strong overall effect of crumb rubber content on viscosity and softening point over the full range from 10% to 35% mainly arises from the contrast between low and high dosage levels. Within each segmented range, the contribution of crumb rubber content becomes more moderate, which supports the use of the segmented orthogonal experimental design to better resolve the effects of the other preparation parameters.

To verify the reliability of these optimized combinations, parallel tests were conducted for K_1_ and K_2_, and the results are shown in Table 9.

Based on the weighted scoring formulas, the scores for the aforementioned 18 orthogonal experimental groups, as well as K_1_ and K_2_, are calculated. Detailed weighting analysis is provided in Table A4. In the low-content group, groups 5 and 9 achieve the highest total scores, while in the high-content group, groups 13 and 16 perform best. Combined with the optimized combinations of K_1_ and K_2_, six comparative samples are formed, as shown in Table 10. Analysis reveals that although groups 5 and 9 have high total scores, their softening points are relatively low, and the 15–20% crumb rubber content offers insufficient raw material cost advantages. K_2_ shows superior road performance in softening point, ductility, and elastic recovery, while its 30% crumb rubber content substantially reduces raw material cost. Although its viscosity was relatively high, it remains within controllable limits and can be further reduced with warm mix additives.

In summary, the optimal preparation process for rubber-modified asphalt was determined as K_2_: aromatic oil 5%, crumb rubber 30%, shear temperature 220 °C, shear time 120 min, and reaction time 90 min. On this basis, various dosages of warm mix additives were incorporated to further evaluate physical and rheological properties.

### 3.2. Physical Property Analysis

**Figure 2:**
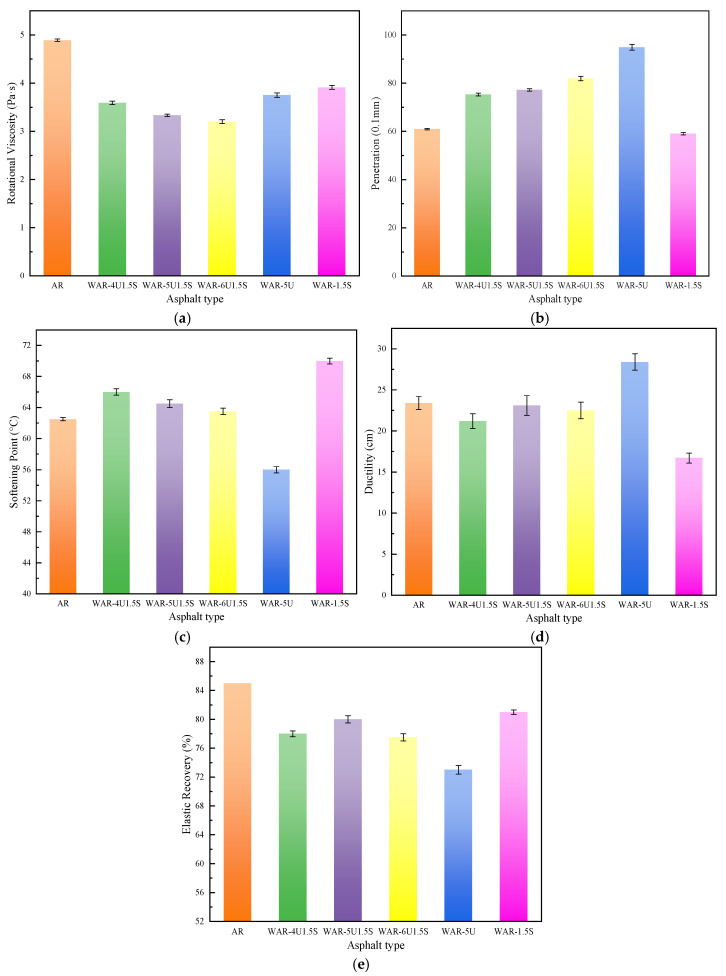
Physical properties of all types of asphalt: (**a**) rotational viscosity; (**b**) penetration; (**c**) softening point; (**d**) ductility; and (**e**) elastic recovery.

Rotational viscosity, penetration, softening point, ductility, and elastic recovery tests were conducted for WAR with different additive dosages. The results are shown in Figure 2.

Figure 2a shows the effect of warm mix additives on the viscosity of asphalt. The result demonstrates a significant viscosity reduction in AR with the addition of warm mix additives. Furthermore, the combined use of UWM and Sasobit produces a larger viscosity reduction than either additive alone, confirming their synergistic effect. With a fixed Sasobit dosage, increasing UWM dosage leads to further viscosity reduction but with a diminishing rate, indicating that surface-active components of UWM approach dispersion saturation in asphalt. Beyond 5%, the viscosity reducing efficiency weakens, necessitating further optimization of the composite ratio to balance economy and performance. UWM reduces the viscosity of asphalt, while Sasobit also contributes to viscosity reduction. Under composite addition conditions, the two acted synergistically to enhance flowability while maintaining colloidal stability, significantly improving workability.

Figure 2b presents the penetration of asphalt with different warm mix additives. The single addition of UWM markedly increases penetration, demonstrating strong plasticizing and softening effects, while the single addition of Sasobit slightly reduces it. The penetration of the composite system falls between the two extremes, increasing linearly with UWM dosage. However, when UWM exceeds 5%, penetration increases sharply, suggesting the need to limit dosage to avoid the loss of high-temperature rutting resistance. Overall, the two effects balance each other, maintaining penetration within an appropriate range.

Figure 2c illustrates the softening point of asphalt influenced by warm mix additives. The single addition of Sasobit raises the softening point significantly, demonstrating its capacity to enhance high-temperature deformation resistance, while the single addition of UWM reduces the softening point. The composite systems maintain softening points between 63.5 and 66.0 °C, slightly higher than AR, and the values decrease only marginally with increasing UWM content, indicating that the stabilizing effect of Sasobit remains dominant and the composite systems retain high-temperature performance.

Figure 2d shows the distinct effects of different additives on the ductility of asphalt. UWM significantly improves ductility, indicating enhanced plastic deformation capability, whereas Sasobit reduces ductility, reflecting its restrictive crystalline structure. The composite systems maintain ductility near the AR baseline and increase slightly with higher UWM dosage, although the rate of improvement diminishes at higher dosages. The results show that the opposing effects of the two additives can be balanced through formulation to maintain adequate ductility.

Figure 2e depicts the elastic recovery of asphalt affected by different warm mix additives. The single addition of either UWM or Sasobit reduces the elastic recovery, with UWM showing a more significant detrimental effect than Sasobit. In the composite systems, elastic recovery demonstrates a clear non-linear relationship with UWM content, initially increasing then decreasing across the tested dosage range. The combination of 5% UWM and 1.5% Sasobit achieves the highest elastic recovery, indicating an optimal balance between the two effects.

Overall, the warm mix additives demonstrate a multidimensional synergistic regulation of WAR performance. UWM and Sasobit jointly reduce viscosity, expand the construction temperature window, maintain balanced consistency by offsetting the plasticizing and hardening effects, enhance high-temperature stability via Sasobit reinforcement, and improve low-temperature ductility through UWM flexibility. The formulation containing 5% UWM and 1.5% Sasobit achieves the optimal balance among all physical indices, demonstrating maximum viscosity reduction, moderate penetration, stable softening point, adequate ductility, and peak percent recovery. This specific formulation thus presents a comprehensive set of properties suitable for practical application.

### 3.3. DSR Test

#### 3.3.1. Effect of Warm Mix Additives on Complex Modulus $G^{*}$

**Figure 3:**
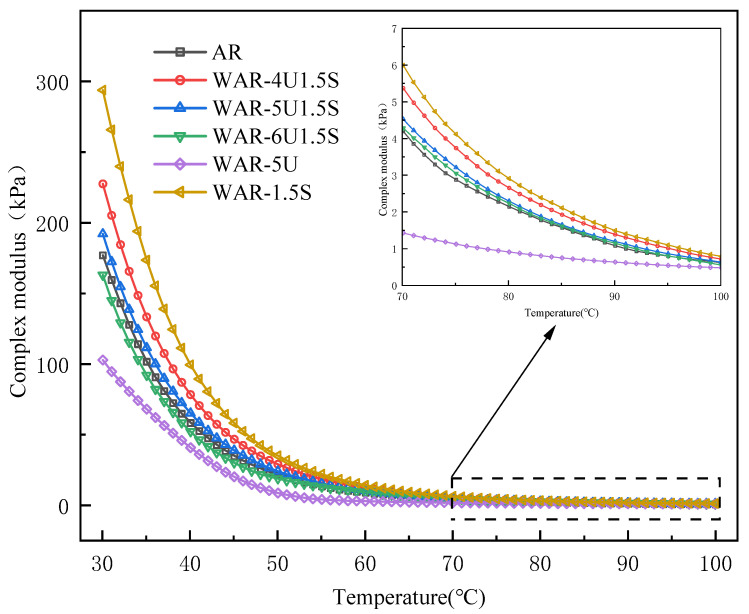
Complex modulus of all types of asphalt.

The complex modulus $G^{*}$ of various asphalt samples as temperature changes is illustrated in Figure 3. The $G^{*}$ of all WAR samples decreases with increasing temperature, which indicates that the stiffness of asphalt is continuously reduced. Moreover, a trend of rapid decay at lower temperatures is observed, followed by a slower decline at higher temperatures within 30–100 °C. The rapid initial decrease is due to the easy disruption of intermolecular forces at low temperature, while the later slowdown corresponds to the stabilized thermal motion of molecules.

In the low to medium temperature range (30–70 °C), WAR-1.5S exhibits the highest $G^{*}$, while WAR-5U remains the lowest. The $G^{*}$ of composite warm mix groups fall between WAR-1.5S and AR, decreasing gradually with increasing UWM dosage. Although the stiffness levels differ, the ranking among samples remains consistent across this temperature interval.

At higher temperatures above 70 °C, the $G^{*}$ of WAR-6U1.5S exceeds AR. When the temperature surpasses the softening point, AR loses its elastic network rapidly, causing accelerated $G^{*}$ decay. In contrast, UWM improves rubber dispersion, while Sasobit provides additional support, allowing WAR-6U1.5S to maintain viscoelastic stability and leading to a higher $G^{*}$ than AR. WAR-5U remains lowest in $G^{*}$ but showed a more gradual decline. As temperature approaches the melting point of Sasobit about 100 °C, the $G^{*}$ of WAR-1.5S decays more rapidly, while the composite systems maintain a slower decline due to the improved dispersion and residual Sasobit support, and the difference between the two groups gradually narrows. These results confirm the synergistic advantages of UWM and Sasobit, where Sasobit enhances medium-temperature stiffness and UWM optimizes high-temperature dispersion, producing balanced viscoelasticity across a wider temperature range and mitigating the temperature sensitivity limitation of single warm mix additives.

#### 3.3.2. Effect of Warm Mix Additives on Phase Angle δ

**Figure 4:**
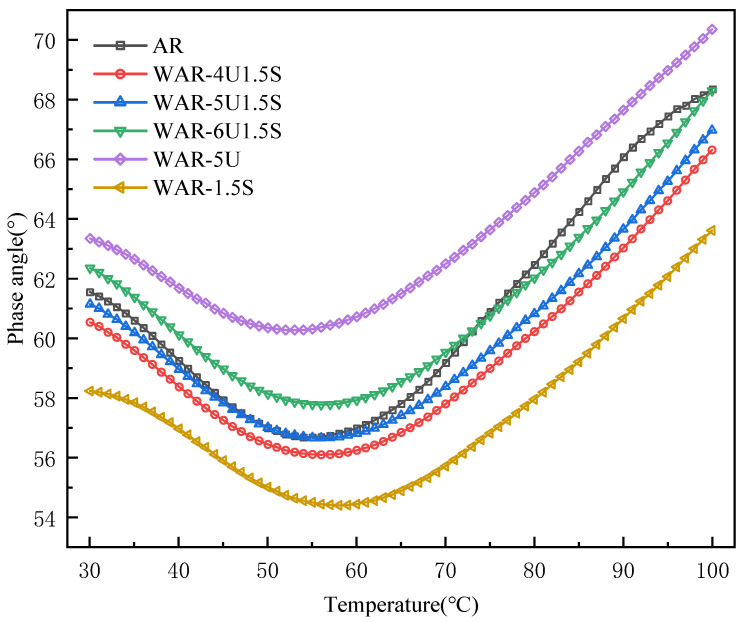
Phase angle of all types of asphalt.

As shown in Figure 4, the δ of all WAR samples exhibits a three-stage evolution: decreasing, plateauing, and then increasing with temperature. The general viscoelastic behavior follows the AR baseline, while UWM and Sasobit mainly influence the magnitude and rate of response.

In the low-temperature range 30–50 °C, the δ of all groups decreases with increasing temperature. WAR-1.5S has the lowest δ due to the synergistic enhancement of elasticity by Sasobit and crumb rubber. The δ of the composite groups fall between WAR-1.5S and AR, as the elasticity-enhancing effect of Sasobit still dominates. WAR-5U, with only UWM, exhibits the largest phase angle due to the weakened elasticity, increasing the viscous contribution.

Between 50 and 60 °C, a stable plateau appears for the δ of all groups with minimal fluctuation. This reflects a temporary equilibrium between molecular thermal motion and rubber elasticity, resulting in a stable ratio of viscous and elastic components. Samples containing Sasobit maintain lower phase angles than AR due to the strengthened elasticity, confirming the performance enhancement by the warm mix additives.

Above 70 °C, the δ increases for all groups as viscosity dominates. After exceeding its softening point, AR experiences a rapid relaxation of the rubber network, losing elasticity and displaying a sharp rise in viscous response, causing the δ of AR to surpass that of all the composite groups. With further temperature increase, molecular forces stabilize and growth slows, resulting in similar values near 100 °C. The composite groups benefit from UWM, which reduces local viscous flow and yields a smoother trend, demonstrating the stabilizing role of warm mix additives at high temperatures.

#### 3.3.3. Effect of Warm Mix Additives on Rutting Factor $G^{*}/\sin\delta$

**Figure 5:**
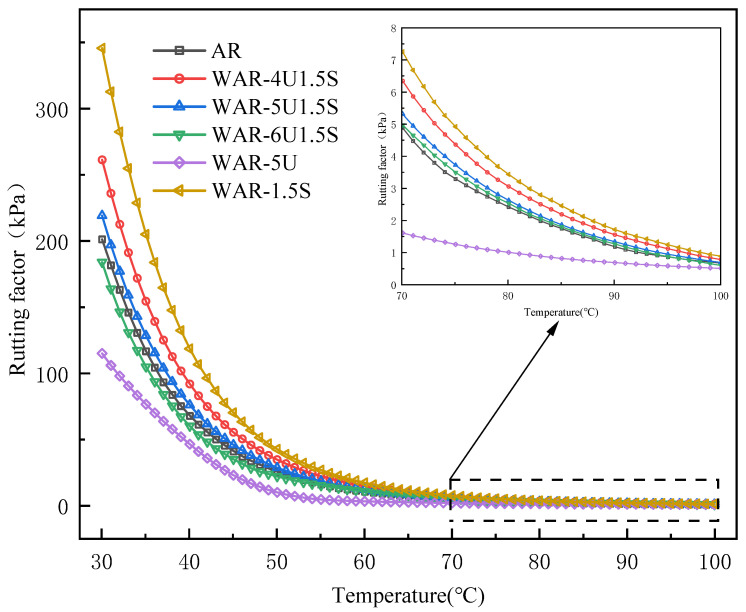
Rutting factor of all types of asphalt.

As shown in Figure 5, the $G^{*}/\sin\delta$ of all WAR groups continuously decreases with increasing temperature, consistent with the trend of $G^{*}$, reflecting the intrinsic temperature response of asphalt viscoelasticity. WAR-1.5S consistently maintains the highest rutting factor because of the increased elastic fraction and limited viscous flow, leading to superior high-temperature deformation resistance. The $G^{*}/\sin\delta$ of the composite groups generally fall between WAR-1.5S and AR, although the reinforcement effects are weaker than that of the Sasobit only group, and performance still improves compared with AR, confirming that the combined modification of UWM and Sasobit effectively enhances the high-temperature stability. WAR-5U has the lowest rutting factor since it relies solely on the improved dispersion of UWM without the structural reinforcement of Sasobit, making the asphalt prone to irreversible flow at high temperatures. Overall, the variation in the rutting factor clearly reveals the synergistic effect of the two additives in regulating the high-temperature performance of asphalt, closely related to the temperature response characteristics of asphalt viscoelasticity, providing a direct reference for optimization.

### 3.4. MSCR Test

**Figure 6:**
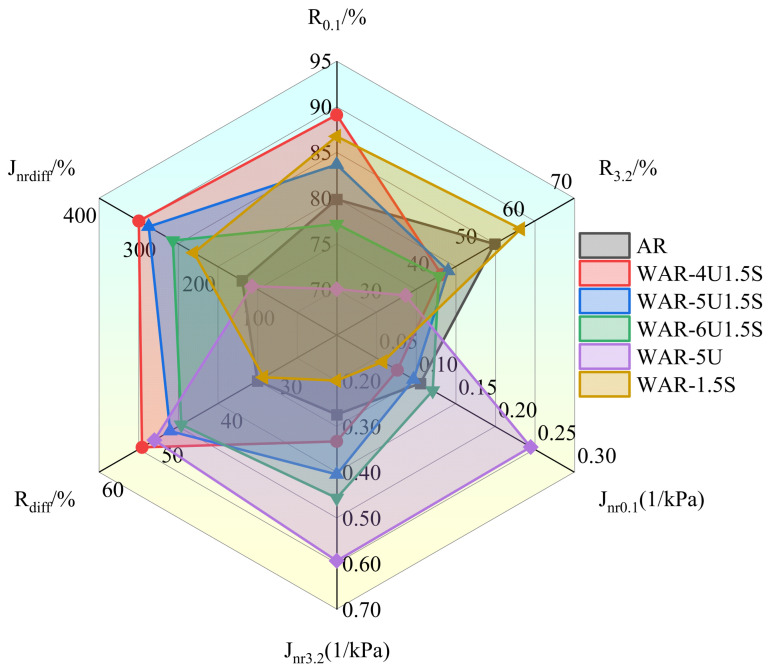
MSCR results of all types of asphalt.

The MSCR test results for WAR are visualized in Figure 6. For all asphalt samples, percent recovery R at 3.2 kPa is much lower than at 0.1 kPa, while non-recoverable creep compliance J_nr_ is higher, indicating that increased stress leads to more permanent deformation, consistent with the viscoelastic response characteristics of asphalt under different stress levels. WAR-1.5S demonstrates the best high-temperature performance, achieving the highest R and lowest J_nr_ at both stress levels. Its J_nrdiff_ and R_diff_ are also the smallest, demonstrating that the incorporation of Sasobit reduces stress sensitivity and improves colloidal rigidity.

WAR-5U exhibits the poorest high-temperature properties. It has the lowest R and highest J_nr_, with significant performance degradation as stress increases. Although R_diff_ is slightly lower than WAR-4U1.5S, it remains higher than AR and WAR-1.5S. This result confirms that the single modification with UWM alone is insufficient to develop adequate elastic recovery and stress resistance compared to systems containing Sasobit, leading to substantially greater accumulation of permanent deformation.

The composite systems demonstrate a balanced performance in high-temperature properties, achieving intermediate values of R and J_nr_ at both stress levels, along with moderate stress sensitivity. Overall, the composite systems maintain more stable and comprehensive MSCR indicators than the single-additive samples, reflecting the combined contribution of both modifiers.

This consistent performance pattern aligns with DSR results, where groups with higher R and lower J_nr_ also exhibit higher $G^{*}$ and smaller δ, reinforcing the mechanism by which warm mix additives regulate asphalt viscoelasticity and providing a quantitative basis for formulation optimization.

### 3.5. BBR Test

**Figure 7:**
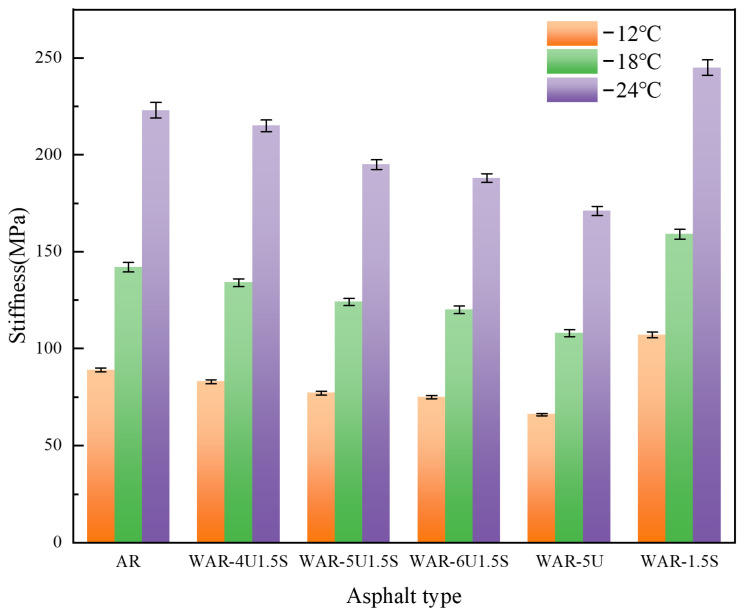
Stiffness of all types of asphalt.

As shown in Figure 7, all WAR samples satisfy the specification criteria of creep stiffness S ≤ 300 MPa and creep rate m-value ≥ 0.3, and exhibit the typical viscoelastic behavior of increasing S and decreasing m-value with falling temperature. The composite-modified groups outperform AR across all temperatures.

For creep stiffness S in Figure 7, the composite groups maintain lower S than AR, with the value decreasing and then stabilizing as the UWM dosage increases. The Sasobit only group produces the highest S, which is associated with its low-temperature stiffening. The incorporation of UWM, however, mitigates this effect by reducing local stress concentrations, thereby mitigating the rise in stiffness. This balancing effect is most pronounced at the 5% UWM dosage.

**Figure 8:**
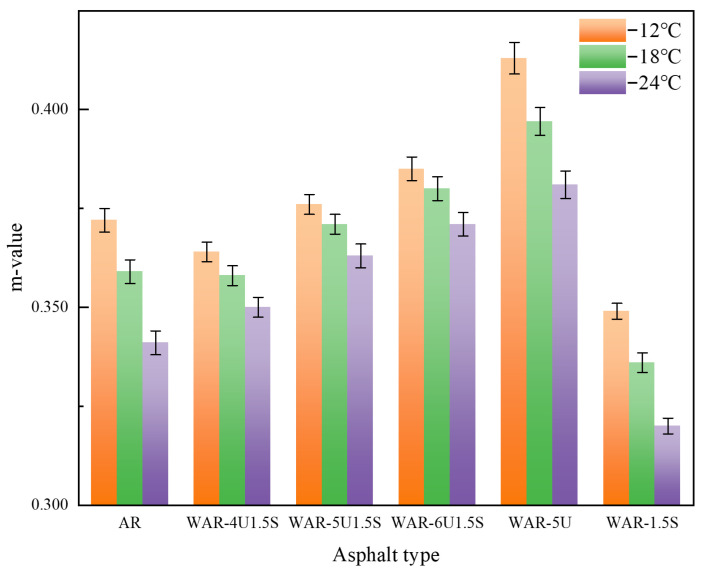
The m-value of all types of asphalt.

As shown in Figure 8, the trend of the m-value further confirms the advantages of composite modification. The m-values of the composite groups are overall better than that of AR, increasing sharply with UWM content before plateauing. UWM enhances the stress relaxation capability and improves the deformation capacity at low temperatures, leading to a higher m-value. In contrast, WAR-1.5S exhibits the lowest m-value and a more restricted deformation response, which increases its brittleness. This is directly related to the characteristic of Sasobit reducing asphalt ductility. The composite systems mitigate this limitation, maintaining a higher m-value and better flexibility.

All samples meet specification requirements, consistent with the optimized parameters determined by the orthogonal experiment. Firstly, aromatic oil, compatible with asphalt components, reduces low-temperature stiffness while enhancing rubber swelling and dispersion. Secondly, the high shear temperature and extended shear time ensure thorough mixing of the components. The high-temperature environment accelerates the desulfurization and degradation of crumb rubber, while prolonged shearing ensures uniform distribution of crumb rubber within the asphalt matrix, preventing property fluctuations. The combined effects of component optimization and process control establish a strong foundation for good low-temperature performance and enable the full benefits of composite modification.

Overall, the composite of UWM and Sasobit has a significant synergistic effect on improving the low-temperature performance of WAR. UWM effectively counteracts the rigidity induced by Sasobit, reducing stiffness and improving deformability. The performance transition from 4% to 5% UWM represents a key improvement zone, while a further increase beyond 5% produces diminishing returns. This confirms that adjusting the formulation of warm mix additives allows accurate control of the low-temperature cracking resistance of WAR. Compared with single-modified systems, the composite approach better balances the characteristics of each component, maintaining warm mix functionality while optimizing viscoelasticity and providing essential experimental support for the formulation design of modified asphalt.

## 4. Conclusions

This study systematically investigates the preparation and unaged performance of WAR. Through a segmented orthogonal experimental design, the optimal preparation parameters of AR are determined, and on this basis, a composite warm mix system combining UWM and Sasobit is constructed. The physical and rheological properties of the system are comprehensively evaluated. The main conclusions are as follows.

1. A five-factor, three-level segmented orthogonal experimental design is employed, and range analysis together with a weighted scoring method is applied, using the key performance indices of AR as the evaluation criteria. The optimal preparation parameters are determined as aromatic oil 5%, crumb rubber 30%, shear temperature 220 °C, shear time 120 min, and reaction time 90 min. These parameters ensure the adequate dispersion and swelling of the crumb rubber and provide a stable baseline for subsequent warm mix modification.
2. Physical tests, including rotational viscosity, penetration, softening point, ductility, and elastic recovery, are used to compare the performance of the composite warm mix system with single-additive warm mix and AR samples. The results show that WAR-5U1.5S exhibits the best comprehensive performance. The composite system achieves a substantial viscosity reduction while maintaining adequate high–low-temperature properties, outperforming both UWM only and Sasobit only binders in overall balance.
3. Rheological tests indicate that the composite warm mix system significantly improves high-temperature performance. Compared with AR, the composite WAR samples exhibit more stable stiffness and phase angle δ evolution in the DSR test, confirming their enhanced high-temperature viscoelastic stability. The composite groups also show higher percent recovery R and lower non-recoverable creep compliance J_nr_ in the MSCR test, reflecting the improved resistance to permanent deformation.
4. BBR test results confirm that all WAR samples satisfy the low-temperature specification requirements. The composite systems show lower creep stiffness S and higher m-values than AR, indicating the improved relaxation capability and cracking resistance. Overall, the composite system is shown to balance high- and low-temperature properties at the binder level and provides a feasible approach for developing WAR. This work focuses on unaged binders, and aging-related long-term performance will be investigated in future work using RTFOT and PAV aging tests.

## Figures and Tables

**Table 1:** Main properties of base asphalt.

Item	Unit	Results	Specifications
Penetration (at 25 °C)	dmm	65.1	60–80
Softening point	°C	47.0	≥46
Ductility (at 10 °C)	cm	30.8	≥20
Viscosity (at 135 °C)	Pa·s	0.86	—
Viscosity (at 145 °C)	Pa·s	0.58	—

**Table 2:** Technical indicators of crumb rubber.

Item	Unit	Results
Heating loss	%	0.62
Ash	%	8.75
Carbon	%	30.51
Iron	%	0.029
Fiber	%	0
Residue on sieve	%	0.014
Bulk density	kg/m^3^	314

**Table 3:** Technical indicators of aromatic oil.

Item	Unit	Results
Color	—	Ink green
Density (at 20 °C)	g/cm^3^	0.990–1.020
Flash point	°C	≥200
Viscosity, dynamic (at 100 °C)	mm^2^/s	15–22
Aniline point	°C	≤40
Sulfur	%	≤5.5
Water	%	≤0.1
Ash	%	≤0.01

**Table 4:** Basic properties of WMA additives.

Properties	UWM	Sasobit
Ingredients	Rubber-derived components and surfactant components	Solid saturated hydrocarbons
Physical state	Paste	Solid
Color	Dark brown	Milky-white
Bulk density	0.983 g/cm^3^	0.621 g/cm^3^
Flash point	>260 °C	N/A
Melting point	N/A	100–110 °C
Solubility in water	Partially soluble	Insoluble
Appearance	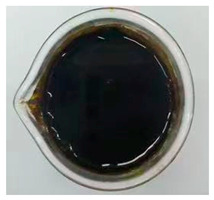	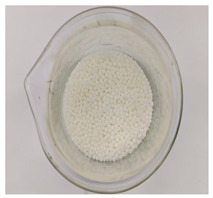

**Table 5:** Orthogonal experimental design scheme.

Number	Orthogonal Factors				
	Factor A (Aromatic Oil/%)	Factor B (Crumb Rubber/%) ^1^	Factor C (Shearing Temperature/°C)	Factor D (Shearing Time/min)	Factor E (Reaction Time/min)
1	4	10	180	60	30
2	4	15	200	90	90
3	4	20	220	120	60
4	5	10	220	120	60
5	5	15	180	90	30
6	5	20	200	60	90
7	6	10	200	90	30
8	6	15	220	60	60
9	6	20	180	120	90
10	4	25	180	60	30
11	4	30	200	90	90
12	4	35	220	120	60
13	5	25	220	120	60
14	5	30	180	90	30
15	5	35	200	60	90
16	6	25	200	90	30
17	6	30	220	60	60
18	6	35	180	120	90

^1^ Factor B was divided into 10~20% (low) and 25–35% (high), with segmented orthogonal experimental design; other factors (A, C, D, E) each had three levels, corresponding to table values.

**Table 6:** Abbreviations and components of asphalt binders investigated.

Abbreviation	Components
AR	Asphalt rubber
WAR-4U1.5S	Warm asphalt rubber with 4% UWM + 1.5% Sasobit
WAR-5U1.5S	Warm asphalt rubber with 5% UWM + 1.5% Sasobit
WAR-6U1.5S	Warm asphalt rubber with 6% UWM + 1.5% Sasobit
WAR-5U	Warm asphalt rubber with 5% UWM only
WAR-1.5S	Warm asphalt rubber with 1.5% Sasobit only

**Table 7:** Results of one-way ANOVA of factors A–E on rotational viscosity and softening point.

Factor	df_between_	df_within_	F (Viscosity)	p (Viscosity)	F (Softening Point)	p (Softening Point)
A	2	15	0.07	0.928	0.26	0.777
B	5	12	14.77	<0.001	12.17	<0.001
C	2	15	0.3	0.744	0.49	0.62
D	2	15	0.17	0.847	0.04	0.96
E	2	15	0.99	0.394	0.83	0.453

**Table 8:** Results of one-way ANOVA of crumb rubber content in low- and high-content groups.

Response	Group	df_between_	df_within_	F	p
Rotational viscosity	Low-content	2	6	12.29	0.008
Rotational viscosity	High-content	2	6	4.04	0.078
Softening point	Low-content	2	6	1.33	0.332
Softening point	High-content	2	6	2.25	0.187

**Table 9:** Test results of K_1_ and K_2_.

Number	Performance Indicators				
	Rotational Viscosity (Pa·s)	Penetration (0.1 mm)	Softening Point (°C)	Ductility (cm)	Elastic Recovery (%)
K1	2.28	58.7	58.0	15.0	76
K2	4.89	60.9	62.5	23.4	85

**Table 10:** Optimal parameter combination.

Number	Aromatic Oil (%)	Crumb Rubber (%)	Shearing Temperature (°C)	Shearing Time (min)	Reaction Time (min)	Total Score
5	5	15	180	90	30	59.4
9	6	20	180	120	90	59.4
13	5	25	220	120	60	51.6
16	6	25	200	90	30	49.9
K_1_	5	20	180	120	30	53.8
K_2_	5	30	220	120	90	54.9

## Data Availability

The raw data, models, and code that support the findings of this study are available from the corresponding author upon reasonable request.

## Appendix A

**Table A1:** Orthogonal experimental results.

Number	Performance Indicators				
	Rotational Viscosity (Pa·s)	Penetration (0.1 mm)	Softening Point (°C)	Ductility (cm)	Elastic Recovery (%)
1	1.71	58.3	56.5	14.8	80
2	1.79	52.7	57.0	14.1	78
3	2.25	56.5	58.5	17.0	77
4	1.46	61.2	55.5	15.9	78
5	1.82	57.1	59.0	14.2	79
6	2.38	52.3	58.5	13.6	73
7	1.53	58.6	55.5	12.5	74
8	1.85	63.9	54.5	18.7	74
9	1.93	59.9	56.5	20.7	78
10	4.51	40.5	63.5	13.0	79
11	5.71	47.8	63.5	15.5	79
12	4.42	53.0	62.0	17.5	77
13	2.98	51.0	60.0	16.0	77
14	6.01	40.0	62.0	15.6	81
15	8.82	45.5	67.0	14.5	81
16	3.51	52.7	61.0	16.0	77
17	5.18	56.6	61.0	16.8	78
18	7.35	50.2	65.0	17.5	80

**Table A2:** Results of range analysis for the low crumb rubber content group (10–20%).

Indicators	Parameters	A	B	C	D	E	Influence Degree
Rotational viscosity (Pa·s)	k_1_	1.92	1.57	1.82	1.98	1.69	B > E > D > A > C
	k_2_	1.89	1.82	1.90	1.71	1.85	
	k_3_	1.77	2.18	1.85	1.89	2.04	
	R	0.15	0.61	0.08	0.27	0.35	
Penetration (0.1 mm)	k_1_	55.8	59.4	58.4	58.2	58.0	C > A > E > B > D
	k_2_	56.9	57.9	54.5	56.1	60.5	
	k_3_	60.8	56.2	60.5	59.2	56.6	
	R	5.0	3.2	6.0	3.1	3.9	
Softening point (°C)	k_1_	57.3	55.8	57.3	56.5	57.0	A > B > C = E > D
	k_2_	57.6	56.8	57.0	57.1	56.2	
	k_3_	55.5	57.8	56.2	56.8	57.3	
	R	2.1	2.0	1.1	0.6	1.1	
Ductility (cm)	k_1_	15.3	14.4	16.6	15.7	13.8	D > C > A = B > E
	k_2_	14.6	15.7	13.4	13.6	15.1	
	k_3_	17.3	17.1	17.2	17.9	16.1	
	R	2.7	2.7	3.8	4.3	2.3	
Elastic recovery (%)	k_1_	78.3	77.3	79.0	75.7	77.7	C > A > D > E > B
	k_2_	76.7	77.0	75.0	77.0	76.3	
	k_3_	75.3	76.0	76.3	77.7	76.3	
	R	3.0	1.3	4.0	2.0	1.4	

**Table A3:** Results of range analysis for the high crumb rubber content group (25–35%).

Indicators	Parameters	A	B	C	D	E	Influence Degree
Rotational viscosity (Pa·s)	k_1_(k_4_)	4.88	3.63	5.96	6.17	4.68	B > E > C > D > A
	k_2_(k_5_)	5.90	5.63	6.01	5.08	4.16	
	k_3_(k_6_)	5.35	6.86	4.16	4.88	7.29	
	R	1.02	3.23	1.85	1.29	3.13	
Penetration (0.1 mm)	k_1_(k_4_)	47.1	48.1	43.6	47.5	44.4	C > E > A > D > B
	k_2_(k_5_)	45.5	48.2	48.7	46.9	53.5	
	k_3_(k_6_)	53.2	49.6	53.5	51.4	47.8	
	R	7.7	1.5	9.9	4.5	9.1	
Softening point (°C)	k_1_(k_4_)	63.0	61.5	63.5	63.8	62.2	E > B > C > D > A
	k_2_(k_5_)	63.0	62.2	63.8	62.2	61.0	
	k_3_(k_6_)	62.3	64.7	61.0	62.3	65.2	
	R	0.7	3.2	2.8	1.6	4.2	
Ductility (cm)	k_1_(k_4_)	15.3	15.0	15.4	14.8	14.9	D > E > A = B = C
	k_2_(k_5_)	15.4	16.0	15.3	15.7	16.8	
	k_3_(k_6_)	16.8	16.5	16.8	17.0	15.8	
	R	1.5	1.5	1.5	2.2	1.9	
Elastic recovery (%)	k_1_(k_4_)	78.3	77.7	80.0	79.3	79.0	C = E > B > A > D
	k_2_(k_5_)	79.7	79.3	79.0	79.0	77.3	
	k_3_(k_6_)	78.3	79.3	77.3	78.0	80.0	
	R	1.4	1.6	2.7	1.3	2.7	

**Table A4:** Results of weighting analysis.

Number	Crumb Rubber Content	Rotational Viscosity Score	Penetration Score	Softening Point Score	Ductility Score	Elastic Recovery Score	Total Socre
1	10%	26.3	9.6	9.8	3.2	6.7	55.6
2	15%	28.1	8.2	10.5	2.7	5.3	54.8
3	20%	25.0	9.1	12.8	4.7	4.7	56.3
4	10%	30.3	9.7	8.3	3.9	5.3	57.5
5	15%	27.9	9.3	13.5	2.8	6.0	59.4
6	20%	24.1	8.1	12.8	2.4	1.3	48.7
7	10%	29.8	9.7	8.3	1.7	2.7	52.1
8	15%	27.7	9.0	6.8	5.8	2.7	52.0
9	20%	27.1	10.0	9.8	7.1	5.3	59.4
10	25%	9.9	5.1	20.3	2.0	6.0	43.4
11	30%	1.9	7.0	20.3	3.7	6.0	38.9
12	35%	10.5	8.3	18.0	5.0	4.7	46.5
13	25%	20.1	7.8	15.0	4.0	4.7	51.6
14	30%	0.0	5.0	18.0	3.7	7.3	34.1
15	35%	0.0	6.4	25.5	3.0	7.3	42.2
16	25%	16.6	8.2	16.5	4.0	4.7	49.9
17	30%	5.5	9.2	16.5	4.5	5.3	41.0
18	35%	0.0	7.6	22.5	5.0	6.7	41.7
K_1_	20%	24.8	9.7	12.0	3.3	4.0	53.8
K_2_	30%	7.4	9.8	18.8	8.9	10.0	54.9
